# Intestinal Calculi: Causes of Error in Renal Radiography

**Published:** 1913-02

**Authors:** 


Intestinal Calculi: Causes of Error in Renal Radiography.—Rochet, Gayet and Arcelin of Lyon, ibid., illustrate various puzzling cases. Renal calculi of uric acid and calcium oxalate may have either as a nucleus so that there may be a dark central mass with a light umbra, or vice versa. They may be found anywhere between the eleventh rib and the iliac crest, or even outside these limits and the mobility or fixity of the calculus is not diagnostic. Calculi of the appendix or colon may simulate renal calculus. Fruit pits, however, are much lighter than calculi, though the differentiation may become impossible as time elapses, from the deposition of lime salts.

